# miR-7 reverses the resistance to BRAFi in melanoma by targeting EGFR/IGF-1R/CRAF and inhibiting the MAPK and PI3K/AKT signaling pathways

**DOI:** 10.18632/oncotarget.10669

**Published:** 2016-07-18

**Authors:** Xiaoyan Sun, Jun Li, Yanhong Sun, Yi Zhang, Liyun Dong, Chen Shen, Liu Yang, Ming Yang, Yan Li, Guanxin Shen, Yating Tu, Juan Tao

**Affiliations:** ^1^ Department of Dermatology, Union Hospital, Tongji Medical College, Huazhong University of Science and Technology (HUST), Wuhan 430022, China; ^2^ Department of Dermatology, Wuhan Central Hospital, Wuhan 430014, China; ^3^ Department of Immunology, Tongji Medical College, HUST, Wuhan 430030, China

**Keywords:** miRNA-7, melanoma, resistance, BRAF inhibitor

## Abstract

MicroRNAs (miRNAs) are attractive therapeutic targets for various therapy-resistant tumors. However, the association between miRNA and BRAF inhibitor resistance in melanoma remains to be elucidated. We used microarray analysis to comprehensively study the miRNA expression profiling of vemurafenib resistant (VemR) A375 melanoma cells in relation to parental A375 melanoma cells. MicroRNA-7 (miR-7) was identified to be the most significantly down-regulated miRNA in VemR A375 melanoma cells. We also found that miR-7 was down-regulated in Mel-CVR cells (vemurafenib resistant Mel-CV melanoma cells). Reestablishment of miR-7 expression could reverse the resistance of both cells to vemurafenib. We showed that epidermal growth factor receptor (EGFR), insulin-like growth factor-1 receptor (IGF-1R) and CRAF were over-expressed in VemR A375 melanoma cells. Introduction of miR-7 mimics could markedly decrease the expressions of EGFR, IGF-1R and CRAF and further suppressed the activation of MAPK and PI3K/AKT pathway in VemR A375 melanoma cells. Furthermore, tumor growth was inhibited in an *in vivo* murine VemR A375 melanoma tumor model transfected with miR-7 mimics. Collectively, our study demonstrated that miR-7 could reverse the resistance to BRAF inhibitors in certain vemurafenib resistant melanoma cell lines. It could advance the field and provide the basis for further studies in BRAF inhibitor resistance in melanoma.

## INTRODUCTION

Constitutively activating mutations in BRAF are found in a large proportion of metastatic melanomas [1]. Although selective small molecular inhibitors targeting this key genetic mutation, e.g., BRAF inhibitors (BRAFi) vemurafenib and dabrafenib, have demonstrated high initial responses [2-4], acquired drug resistance is almost universal and relapse occurs in almost all patients [5]. Up to 70% of resistance to BRAFi were identified through MAPK reactivation with RAS and MEK mutations, as well as mutant BRAF amplification or alternative splicing being the most prevalent [6-8, 17]. Upregulating genetic mutations in PI3K/AKT pathway as second core resistance mechanism also play an important role in acquired BRAFi resistance [9, 10]. Therefore, understanding how genetic mutations shape the resistance to BRAFi and exploring how gene modulations (e.g., RNA interference with microRNA and small interfering RNA) reverse BRAFi resistance will become more important.

MicroRNA (miRNA), a 20-22 nucleotide noncoding RNA, is capable of modulating gene expression through imperfect base-pairing with specific sequences in the 3′ untranslated regions (UTRs) of target mRNAs, causing either mRNA degradation or translation inhibition [11]. The 3′UTRs of a single target mRNA contains numerous different miRNA binding sites which vice versa enables a single miRNA to bind and fine-tune several target mRNAs with different function simultaneously and alter the activity of various disease-associated signaling pathway. Dysregulation of miRNAs involves in many cellular processes including differentiation, proliferation, apoptosis and metastasis that are important for tumorigenesis [12, 22-24]. Recently, a growing amount of research has revealed that miRNAs may also take part in the resistance of anti-cancer therapeutic agents by regulating some of the resistance-associated signaling pathways in tumor cells. Examples include: inhibition of miR-21 enables to increase gemcitabine sensitivity in cholangiocarcinoma [13] and to suppress the growth of topotecan-recalcitrant MCF-7 cells [14]; miR-203 targets the 3′-UTR of ABL and leads to sensitization of BaF3-BCR/ABL cells to imatinib in chronic myeloid leukemia (CML) [15]; miR-125b attenuated epithelial-mesenchymal transition (EMT), including chemoresistance, migration and stemness in hepatocellular carcinoma (HCC) [16], etc. However, limited miRNA-related research on BRAFi resistance in melanoma has been carried out with few publications in this field.

In this study, we developed and characterized VemR A375 and Mel-CVR cell lines (vemurafenib resistant A375 and Mel-CV melanoma cells, respectively) and established xenografted VemR A375 mice model to investigate whether miRNAs are involved in the role of BRAFi resistance in melanoma. We identified that 17 miRNAs dysregulated in VemR A375 melanoma cells compared to vemurafenib-sensitive parental A375 cells and miR-7 was the most significantly down-regulated miRNA among them. The expression level of miR-7 was also down-regulated in Mel-CVR cells. Reestablishment of miR-7 expression could reverse the resistance to BRAFi in both VemR A375 and Mel-CVR melanoma cells. We also found that epidermal growth factor receptor (EGFR), insulin-like growth factor-1 receptor (IGF-1R) and CRAF were over-expressed in VemR A375 melanoma cells. miR-7 could decrease the expressions of EGFR, IGF-1R and CRAF and further suppressed the activation of MAPK and PI3K/AKT pathways in VemR A375 melanoma cells. Furthermore, tumor growth was inhibited in an *in vivo* murine VemR A375 melanoma tumor model transfected with miR-7 mimics. Our study revealed the potential involvement of miR-7 in BRAFi resistant melanoma growth under some circumstances and raised the possibility that this might be exploited therapeutically.

## RESULTS

### miR-7 is down-regulated in VemR A375 and Mel-CVR melanoma cells and reestablishment of miR-7 expression reverses the resistance to vemurafenib

**Figure 1:**
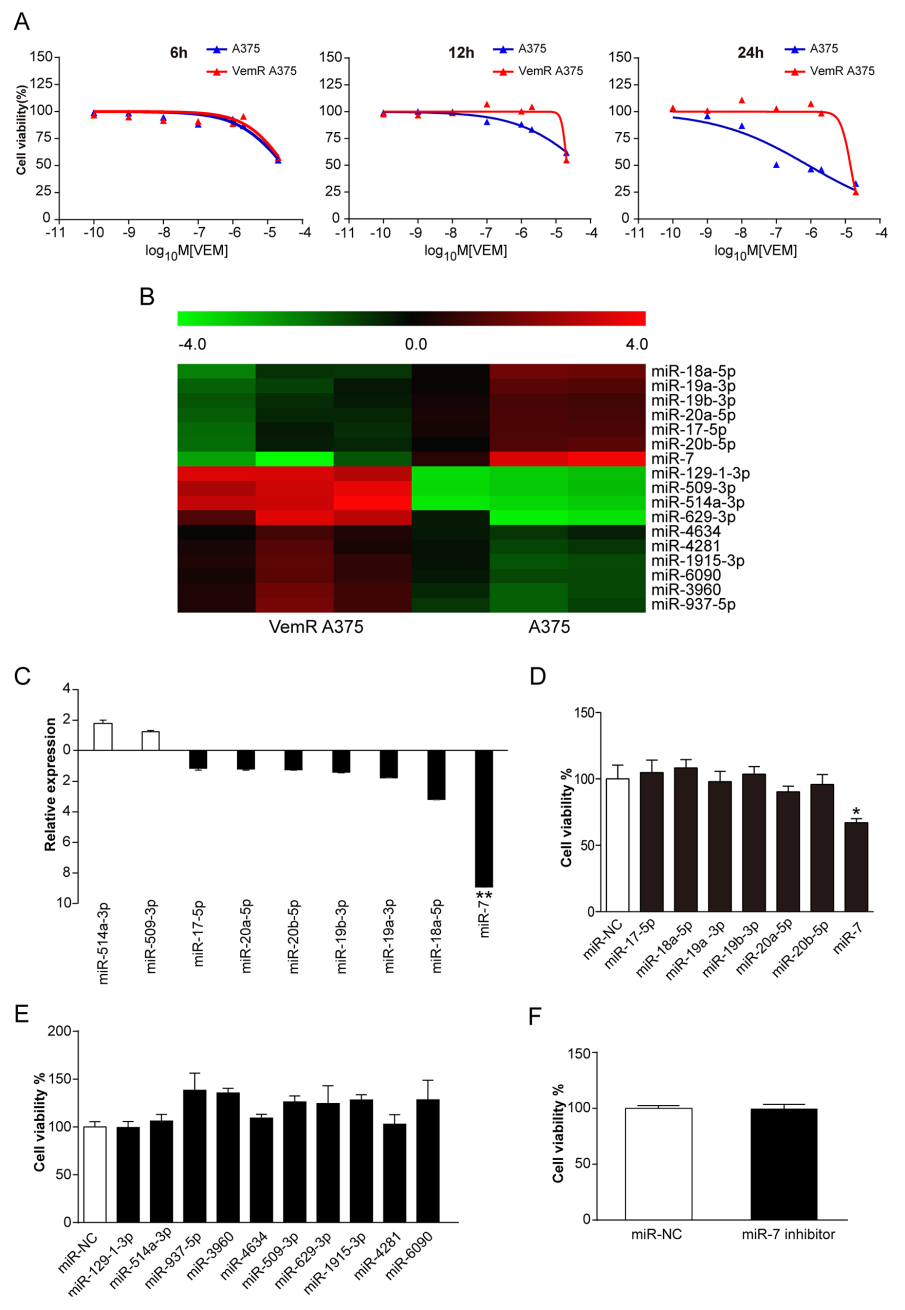
miR-7 is down-regulated in VemR A375 melanoma cells and reestablishment of miR-7 expression sensitizes VemR A375 melanoma cells to vemurafenib **A.** Cell viability of parental A375 melanoma cells (blue) and VemR A375 melanoma cells (red) under different concentrations of vemurafenib treatment was assessed by CCK8 assays for 6, 12 and 24 hrs. **B.** Microarray analysis showing miRNA expression profiles of VemR A375 melanoma cells compared with parental A375 cell controls. **C.** qRT-PCR showing deregulated miRNAs expressions in VemR A375 melanoma cells compared with that in parental A375 melanoma cells. The data were normalized to the level of U6 RNA in each sample. Nine validated up- and down-regulated miRNAs values are shown as the mean ± SD. **D.** Gain-of-function studies of down-regulated miRNAs on VemR cell growth exposed to 2 uM vemurafenib for 72 hrs. **E.** Knock-down studies of up-regulated miRNAs on VemR cell growth exposed to 2 uM vemurafenib for 72 hrs. **F.** Cell viability of parental A375 melanoma cells stably transfected with miR-NC or miR-7 inhibitor exposed to 100 nM vemurafenib for 72 hrs. These experiments were carried out in triplicate and results are shown as the mean ± SD. *p < 0.05, **p < 0.01. VemR: vemurafenib resistant, qRT-PCR: quantitative real-time PCR, miR-NC: microRNA normal control.

VemR A375 and Mel-CVR melanoma cell lines were generated and BRAFi resistance was characterized by measuring cell viability under gradually increased concentrations of vemurafenib treatment (Figure 1A and Supplementary Figure S1A). In order to identify miRNAs that are potentially involved in the underlying mechanisms of vemurafenib resistance, we employed miRNA microarray and determined the miRNAs that differentially expressed between parental A375 and VemR A375 cells. Seventeen miRNAs were found which had 2-folds or greater differences in levels in VemR A375 melanoma cells as compared with parental A375 cells by microarray (Figure 1B and Supplementary Table S1), with 7 down-regulated miRNAs including miR-7 (40.3-fold), miR-18a-5p (5.2-fold), miR-19a-3p (3.6-fold), miR-20b-5p (3.4-fold), miR-17-5p (3.2-fold), miR-20a-5p (3.1-fold), and miR-19b-3p (2.8-fold) and 10 up-regulated miRNAs including miR-514a-3p (116-fold), miR-129-1-3p (87-fold), miR-509-3p (83-fold), miR-629-3p (22-fold), miR-937-5p (4.6-fold), miR-3960 (4.3-fold), miR-1915-3p (3.2-fold), miR-6090 (3.1-fold), miR-4281 (2.6-fold) and miR-4634 (2-fold).

To validate the results of microarray, selected miRNAs differentially expressed in VemR A375 melanoma cells were analyzed by qRT-PCR. Nine of these deregulated miRNAs were confirmed to be either up-regulated (2 miRNAs) or down-regulated (7 miRNAs) with miR-7 being the most significantly down-regulated one (8.9-fold, *p* < 0.01) in 3 independent assays (Figure 1C). We next investigated if these deregulated miRNAs could reverse the resistance to vemurafenib. Synthetic oligonucleotide miRNA mimics of seven down-regulated miRNAs were transfected into VemR A375 cells and then exposed to 2uM vemurafenib. Cell viability of each group was measured 72 hrs after transfection. Compared with miRNA-negative control (miR-NC), VemR A375 cell viability was reduced by 30% (*p* < 0.05) only in miR-7 mimics transfection group while the other six down-regulated miRNA mimics did not show any significant inhibitory effects on VemR cell proliferation (Figure 1D). In addition of its decreased expression in VemR A375 cells, miR-7 was also identified to be down-regulated in Mel-CVR melanoma cells as compared with parental Mel-CV cells by qRT-PCR analysis (*p* < 0.05) (Supplementary Figure S1B). After transfection with miR-7 mimics for 72 hrs, the expression level of miR-7 in Mel-CVR cells was significantly up-regulated (*p* < 0.05) (Supplementary Figure S1C). We next tested the cell viability of Mel-CVR melanoma cells which were transfected with miR-7 mimics and cultured under 2 uM of vemurafenib for 72hrs. Our study showed that the cell viability of Mel-CVR was reduced to 75% (*p* < 0.05) as compared with miR-NC (Supplementary Figure S1D). These results indicated that miR-7 was associated with vemurafenib resistance and reestablishment of miR-7 expression was capable of inhibiting the proliferation of both VemR A375 and Mel-CVR cells and reversing the resistance to vemurafenib. In the meantime we transfected miRNA inhibitors of ten up-regulated miRNAs into VemR A375 cells, and none of these miRNA inhibitors could significantly reduce the cell viability of these cells (Figure 1E).

We next tested if the down-regulation of miR-7 was also involved in acquiring resistance to vemurafenib in parental A375 cells. We found that when treated with 100 nM vemurafenib for 72 hrs, parental A375 cells transfected with miR-7 inhibitor showed no significant differences in proliferation as compared with those cells transfected with miR-NC, which suggested that miR-7 exhibited no inhibitory effect on the proliferation of parental A375 melanoma cells when exposed to BRAFi (Figure 1F).

### miR-7 suppresses the activation of MAPK and PI3K/AKT signaling pathways in VemR A375 cells

**Figure 2:**
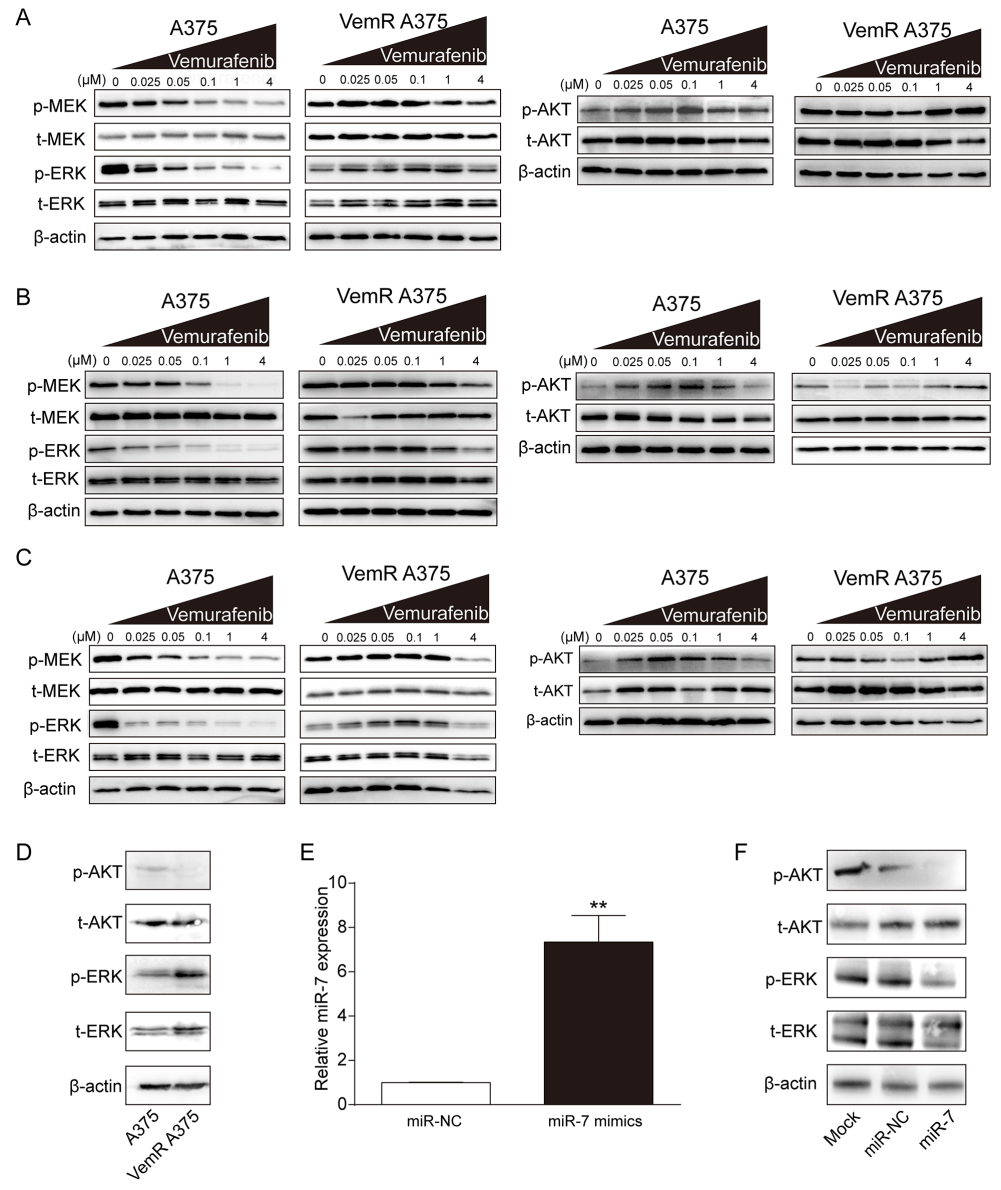
miR-7 suppresses the activation of MAPK and PI3K/AKT pathways in VemR A375 cells **A, B,** and **C.** Expressions of MAPK pathway components and effectors and levels of AKT phosphorylation in parental and VemR A375 melanoma cells treated with the indicated concentrations of vemurafenib for 6, 12 and 24 hrs. **D.** Western blot results of phosphorylated levels of AKT and ERK in parental compared with VemR A375 melanoma cells. **E.** Expression levels of miR-7 in VemR A375 melanoma cells transfected with miR-7 or miR-NC. **F.** Western blot results of p-AKT and p-ERK in VemR A375 melanoma cells transfected with miR-7 or miR-NC. These experiments were carried out in triplicate and results are shown as the mean ± SD. **p < 0.01.

To investigate the molecular basis underlying the potential role of miR-7 in the reversal of acquired resistance to BRAFi, we first analyzed the effect of vemurafenib on MAPK and PI3K/AKT, two of the most important signaling pathways involved in the resistance to BRAFi, in both parental and VemR A375 cells. In the absence of vemurafenib, both parental and VemR A375 melanoma cells showed high levels of phosphorylated MEK (p-MEK) and ERK (p-ERK). The expressions of p-MEK and p-ERK gradually decreased while the phosphorylated AKT (p-AKT) levels were observed to be gradually elevated in parental A375 cells 6, 12 and 24 hrs after treatments with increased concentrations of vemurafenib from 0.025 uM to 1 uM, respectively (Figure 2A, 2B, 2C and Supplementary Figure S2). The results indicated that the activation of PI3K/AKT signaling pathway (increased p-AKT) in parental A375 cells might be an early anti-apoptotic response to BRAF inhibition, which could compensate for the inhibition of MAPK signaling pathway (decreased p-ERK) to maintain the proliferation of parental A375 melanoma cells. On the contrary, vemurafenib had little effect on the expression of p-MEK or p-ERK, even at high concentrations in VemR A375 cells. In addition, p-AKT level was relatively unchanged in VemR A375 cells following vemurafenib treatment (from 0 to 4 uM) (Figure 2A, 2B, 2C and Supplementary Figure S2). Furthermore, we showed the differences of p-ERK and p-AKT levels between the original and the late stage of A375 melanoma cells to elucidate the expression changes of these key molecules and their potential role in the development of vemurafenib resisitance (Figure 2D). During the original stage, A375 melanoma cells were transiently exposed to increasing concentrations of vemurafenib and have not developed resistance to vemurafenib. They are referred to as “parental A375 cells”. Acute BRAF inhibition by vemurafenib led to increased p-AKT but decreased p-ERK levels in parental A375 cells compared with those in VemR A375 cells (Figure 2D), suggesting that activated AKT signaling pathway might rescue the sudden inhibition of MAPK pathway to maintain the survival of parental A375 cells. In contrast, during the late stage, parental A375 melanoma cells were continuously exposed to 2 uM of vemurafenib and evolved into VemR A375 melanoma cells. Upon chronic BRAF inhibition, p-ERK level of VemR A375 cells was restored and even much higher, while p-AKT was lower than those of parental A375 cells (Figure 2D). These data indicated that the proliferation and survival of VemR A375 melanoma cells were mainly dependent on MAPK signal but not AKT activation. The enhanced activity of MAPK and attenuated activity of AKT associated with chronic BRAF inhibition suggests the possible existence of a negative crosstalk between these two pathways.

We next transfected VemR A375 cells with miR-7 mimics or miR-NC to investigate whether miR-7 regulates MAPK and PI3K/AKT signaling pathway in these cells. We first verified a 7.2-fold increase in miR-7 expression after transfected with miR-7 mimics (*p* < 0.01) compared to miR-NC (Figure 2E). Compared with mock and miR-NC, the expressions of p-AKT and p-ERK in VemR A375 cells transfected with miR-7 mimics were significantly decreased (Figure 2F). The results indicated that miR-7 might reverse the resistance to BRAFi in VemR A375 cells by suppressing the activation of both PI3K/AKT and MAPK pathways.

### miR-7 suppresses the expressions of EGFR, IGF-1R and CRAF that are up-regulated in VemR A375 cells

**Figure 3:**
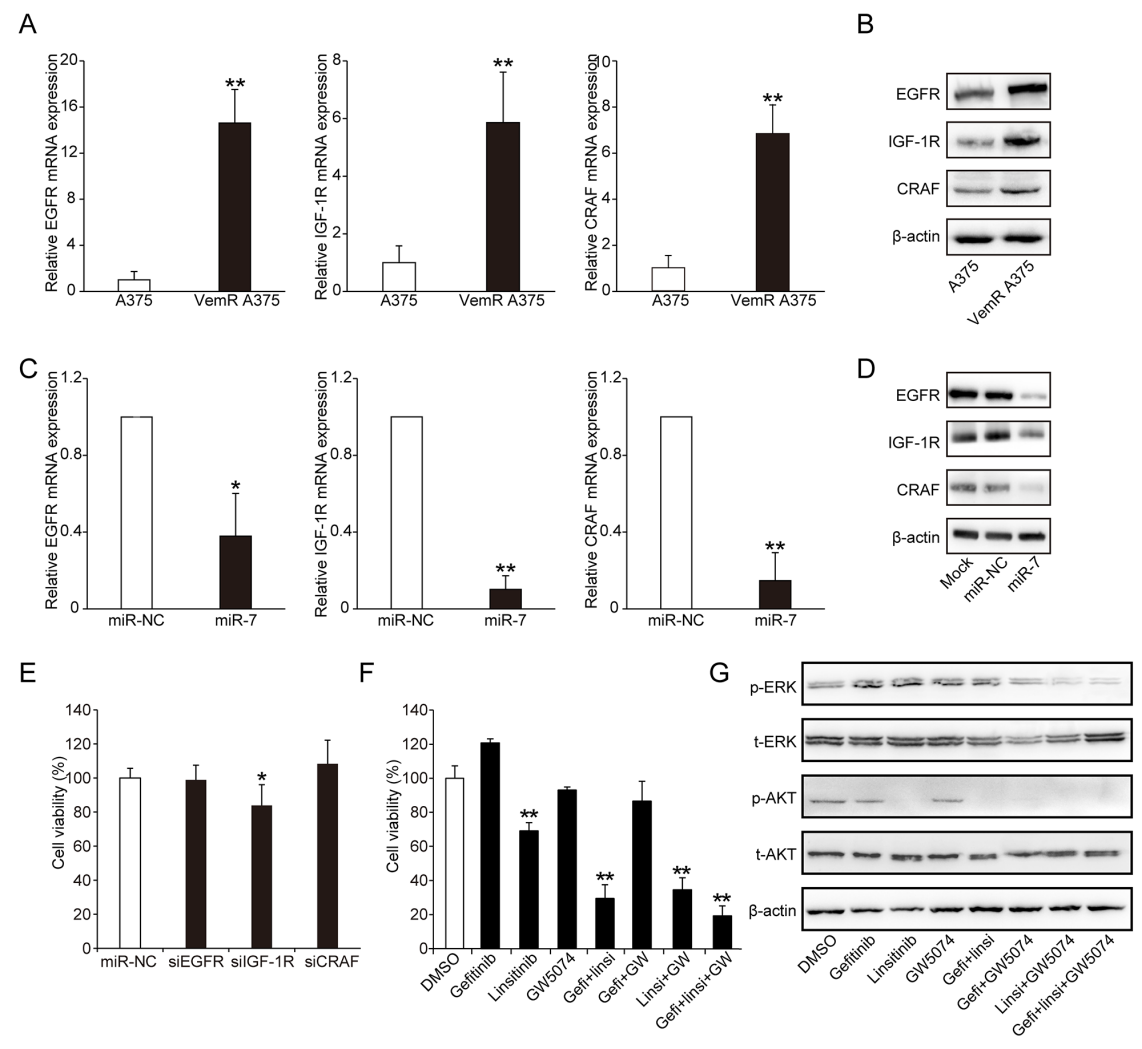
miR-7 suppresses the expressions of EGFR, IGF-1R and CRAF that are up-regulated in VemR melanoma cells The expression levels of EGFR/IGF-1R/CRAF mRNA **A.** and protein **B.** in parental and VemR A375 melanoma cells. The expression levels of EGFR/IGF-1R/CRAF mRNA **C.** and protein **D.** in VemR A375 melanoma cells transfected with miR-NC or miR-7 mimics for 48 hrs. **E.** knock-down studies of up-regulated EGFR/IGF-1R/CRAF on VemR A375 melanoma cell growth exposed to vemurafenib. **F.** Cell viability of VemR A375 melanoma cells treated with gefitinib, linsitinib, GW5074 individually or in different combination with 2 uM vemurafenib for 72 hrs. **G.** The expression levels of p-ERK and p-AKT proteins of VemR A375 melanoma cells treated with gefitinib, linsitinib, GW5074 individually or in different combination with 2 uM vemurafenib for 72 hrs. These experiments were carried out in triplicate and results are shown as the mean ± SD. *p < 0.05, **p < 0.01. EGFR: epidermal growth factor receptor, IGF-1R: insulin-like growth factor-1 receptor, Gefi: gefitinib, Linsi: linsitinib.

To further identify the target genes of miR-7 and investigate how miR-7 regulates the expression of these targets to reverse BRAFi resistance, we first used TargetScan (http://www.targetscan.org/) and PicTar (http://pictar.mdc-berlin.de/) to search for miR-7 target genes. Among these candidate genes, EGFR, IGF-1R, CRAF, AXL and microphthalmia-associated transcription factor (MITF) captured our attention because of their potential role in vemurafenib resistance reported by previous studies. mRNA expressions of EGFR (*p* = 0.0014), IGF-1R (*p* = 0.0014) and CRAF (*p* = 0.0019) were significantly elevated in VemR A375 cells compared with parental A375 cells (Figure 3A). Western blotting results also showed that the protein expressions of EGFR, IGF-1R and CRAF were significantly increased in VemR A375 cells than those in parental A375 cells (Figure 3B). We next transfected VemR A375 cells with miR-7 mimics for 48 hrs and found that not only the mRNA expressions of EGFR (*p* = 0.0402), IGF-1R (*p* = 0.0021) and CRAF (*p* = 0.0097) significantly decreased, the protein levels of these genes were also markedly decreased in these cells (Figure 3C and 3D). However, we failed to show miR-7 directly regulate AXL and MITF expression (data note shown). Therefore, our study indicated that miR-7 could regulate vemurafenib resistance by suppressing the over-expression of EGFR, IGF-1R and CRAF in VemR A375 cells.

### Inhibition of EGFR/IGF-1R/CRAF decreases VemR melanoma cell proliferation by suppressing the activation of MAPK and PI3K/AKT signaling pathways

To verify the potential roles of EGFR, IGF-1R and CRAF in vemurafenib resistance, we first used siRNA-mediated knockdown of EGFR, IGF-1R and CRAF to inhibit their expression in VemR A375 cells and confirmed their suppression effect by Western blot assays (Supplementary Figure S3). Knockdown of IGF-1R gene by transfection of exogenous IGF-1R siRNA resulted in a mild decrease in cell proliferation by 17% in VemR A375 cells 72 hrs after transfection (*p* = 0.0279), suggesting that resistance to vemurafenib is partially attributed to increased IGF-1R expression (Figure 3E). However, knockdown of EGFR and CRAF genes respectively did not exhibit any significant proliferation inhibition (Figure 3E), suggesting that these two genes individually might not play major roles in vemurafenib resistance. We next co-transfected VemR A375 cells with two or three siRNAs of each gene and analyzed the cell proliferation 72 hrs after transfection. However, combined“direct targeting”siRNAs showed no significant inhibitory effect on VemR A375 cell proliferation (Supplementary Figure S4). The “off-target” effect might be due to the interactions among these nucleotide sequences which may weaken the binding abilities to their targets.

We subsequently used small molecular inhibitors of EGFR (gefitinib), IGF-1R (linsitinib), and CRAF (GW5074), as single agent or in combination to further verify whether these genes really participate in BRAFi resistance in VemR A375 melanoma cells. VemR A375 cell growth was suppressed by 31% after linsitinib treatment for 72 hours (*p* < 0.0001) (Figure 3F). Concomitant EGFR and IGF-1R inhibition with gefitinib and linsitinib led to a significant decrease in cell proliferation by 71% (*p* < 0.0001) (Figure 3F). Similar result was observed when inhibiting IGF-1R and CRAF with linsitinib and GW5074 (cell proliferation suppressed by 66%, *p* < 0.0001) (Figure 3F). Although treatment with gefitinib and GW5074 did not significantly inhibit the growth of VemR A375 cells (Figure 3F), combined inhibition with gefitinib and GW5074 could markedly reduce the expressions of p-EKR and p-AKT protein (Figure 3G), which indicated that other signaling pathways might be activated to compensate for the inhibition of MAPK and PI3K/AKT pathways to maintain the proliferation of VemR melanoma cells. Co-administration with gefitinib, linsitinib and GW5074 could achieve the most significant inhibition of cell proliferation in VemR A375 cells (cell proliferation suppressed by 81%, *p* < 0.0001) (Figure 3F), indicating that co-inhibition of EGFR/IGF-1R/CRAF could markedly decrease VemR melanoma cell proliferation.

Since EGFR, IGF-1R and CRAF have been known to be the key components of RTK-RAS-RAF-MEK-ERK (MAPK) and/or PI3K/AKT signaling pathways, we further determined if single or combined inhibition of EGFR, IGF-1R and CRAF could suppress the activation of MAPK and PI3K/AKT signaling in VemR melanoma cells. Treatment with linsitinib for 72 hrs resulted in decreased level of p-AKT, but had no significant effect on the expressions of p-ERK (Figure 3G). Combined inhibition of EGFR and IGF-1R with gefitinib and linsitinib also led to decreased p-AKT, but without any significant inhibition of p-EKR (Figure 3G). Combined inhibition of EGFR and CRAF with gefitinib and GW5047, or combined inhibition of IGF-1R and CRAF with linsitinib and GW5047 for 72 hrs not only reduced p-ERK protein level, but also decreased the expressions of p-AKT (Figure 3G). In addition, co-administration with gefitinib, linsitinib and GW5074 for 72 hrs also reduced p-ERK and p-AKT expressions in VemR A375 cells (Figure 3G). The collective data suggested that co-inhibition of EGFR/IGF-1R/CRAF could decrease VemR A375 melanoma cell proliferation to a certain extent through the suppression of the activation of MAPK and/or PI3K/AKT signaling pathways.

### miR-7 inhibits VemR melanoma tumor growth *in vivo*

**Figure 4:**
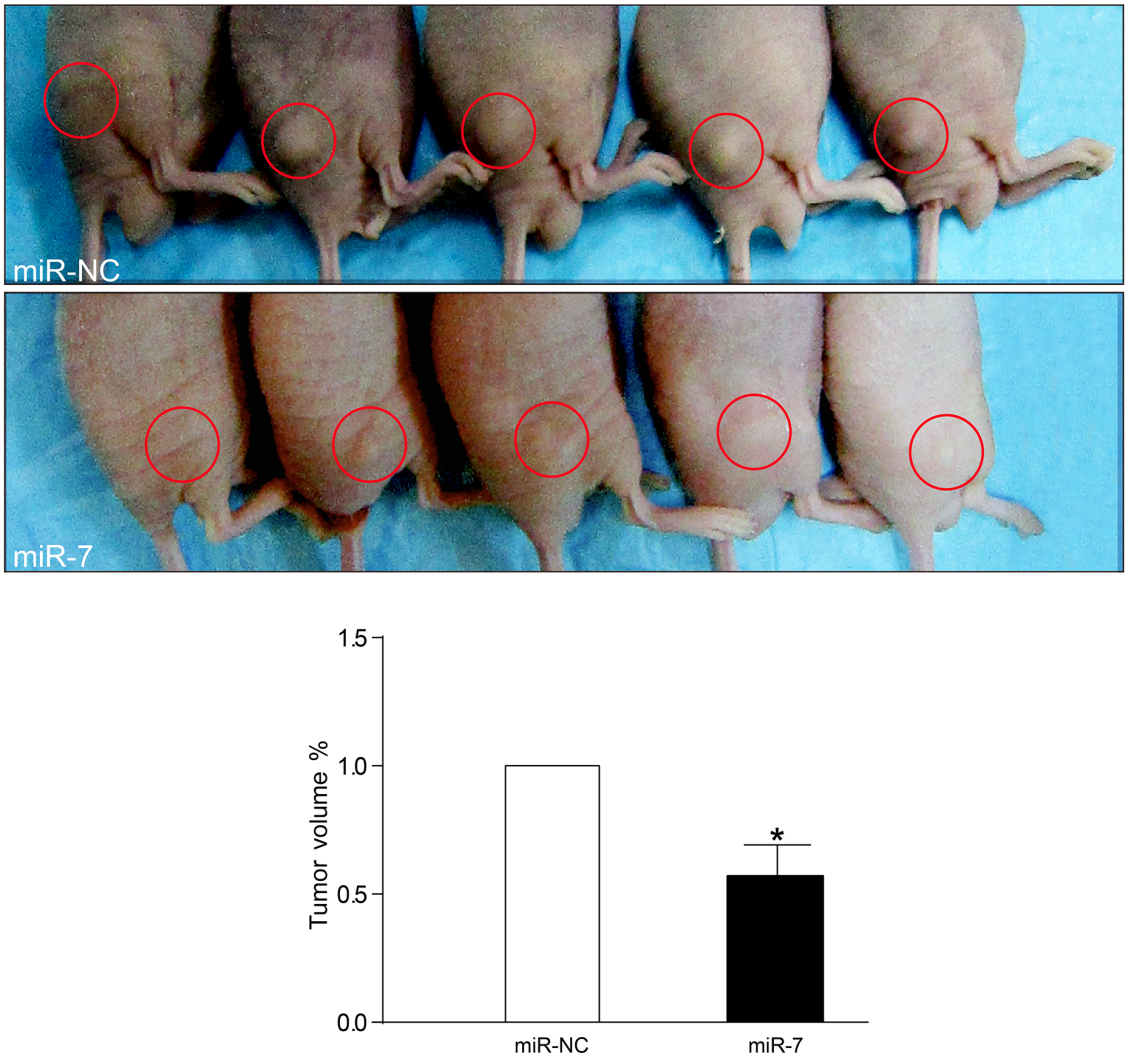
miR-7 inhibits VemR A375 melanoma tumor growth *in vivo* VemR A375 melanoma cells (2 × 10^5^) were transfected with miR-NC or miR-7 mimics and then injected subcutaneously to athymic nude mice (n = 5 for each group). One week later, cohorts of mice from each group were sacrificed to determine the tumor volume. The *in vivo* experiment was repeated twice. Representation (up) and quantification (down) of xenograft tumors formed by subcutaneous injection of VemR A375 melanoma cells stably transfected with miR-NC or miR-7 mimics. *p < 0.05.

To further investigate whether increased levels of miR-7 could inhibit VemR melanoma growth *in vivo*, VemR A375 cells were transfected with miR-7 mimics (VemR A375-miR-7) or miR-NC (VemR A375-miR-NC) to produce subcutaneous tumors in athymic nude mice. Mice were sacrificed 7 days after implantation, tumors were removed and tumor volumes of each mouse were measured. The tumors in VemR A375-miR-7 group grew more slowly than those in VemR A375-miR-NC group with significantly smaller tumor volumes at day 7 post-implantation (Tumor volumes_miR-7_/Tumor volumes_miR-NC_ = 0.5723, *p* = 0.0116) (Figure 4). These data indicated that elevated miR-7 levels in VemR A375 cells markedly suppressed tumor growth.

**Figure 5:**
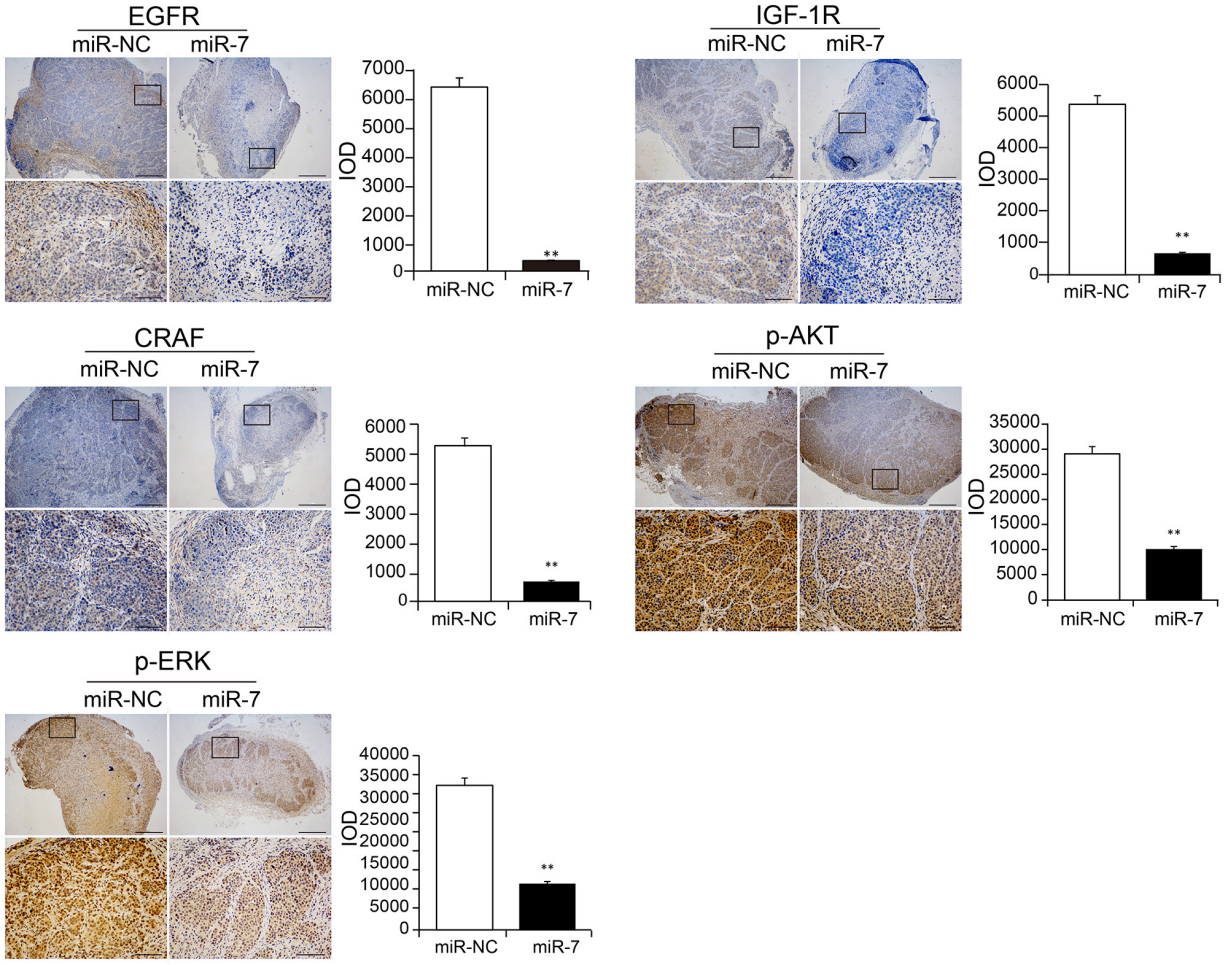
Immunohistochemical staining showing the expressions of EGFR, IGF-1R, CRAF, p-ERK and p-AKT within tumors formed by hypodermic injection of VemR A375 melanoma cells stably transfected with NC or miR-7 mimics Low magnification representation (up, scale bars: 500 mm) and higher magnification representation (down, scale bars: 50 mm) as well as quantification (bar charts) of EGFR, IGF-1R, CRAF, p-ERK and p-AKT determined by automated counting of IOD levels (n = 5 for each group). **p < 0.01.

In the meantime, to verify the underlying mechanism of the inhibitory effect of miR-7 on VemR melanoma tumor growth we also performed the immunohistochemistry (IHC) staining of EGFR, IGF-1R, CRAF, p-ERK and p-AKT and analyzed the total integrated optical density (IOD) of each parameter from both tumor groups. The IOD levels of EGFR, IGF-1R, CRAF, p-ERK and p-AKT in VemR A375-miR-7 group were significantly decreased compared with those in VemR A375-miR-NC group (*p* < 0.01) (Figure 5). These data also suggested that miR-7 could inhibit VemR melanoma cell proliferation through combined inhibition of EGFR/IGF-1R/CRAF expressions, which further decreased the levels of p-ERK and p-AKT in VemR A375 cells.

## DISCUSSION

Malignant melanoma is among the deadliest and treatment-resistant cancers with an increasing incidence rate worldwide. Despite great breakthrough of targeted therapies (e.g., BRAF inhibitors and MEK inhibitors) have revolutionized the treatment strategies for metastatic melanoma, durable clinical responses remain elusive [10]. Several studies have shown that over-expression of EGFR, FGFR3, PDGFRβ, CRAF, and NRAS in melanoma have been associated with BRAFi resistance [6, 10, 18-21]. Moreover, when the hyperactivated MAPK signaling pathway was blocked by BRAFi, enhanced IGF-1R and PI3K/AKT activity contribute to the secondary (acquired) drug resistance [9].

MicroRNAs have recently gained more attention due to their potential roles in tumorigenesis. Some miRNAs have also been shown to be involved in the acquired resistance to different therapies in a variety of tumor types including melanoma [25-31]. However, the comprehensive analysis and research of miRNAs on the resistance to BRAFi in melanoma has been limited. To identify BRAFi resistance-associated microRNAs, we used microarray profiling analysis to comprehensively study VemR A375 melanoma cells in relation to parental A375 melanoma cells. In our study we totally identified 17 miRNAs differentially expressed with statistical significance (≥ 2 fold, *p* < 0.05). Among them miR-7 was found to be the most significantly down-regulated miRNA in VemR A375 melanoma cells. Our studies also demonstrated that reestablishment of miR-7 expression could reverse the resistance to vemurafenib and inhibited resistant A375 melanoma cell growth both *in vitro* and in xenograft tumor models.

The first question is to identify the target genes of miR-7 that participate in the acquired resistance to BRAFi in melanoma. Some miR-7 associated cancer research have also shown that miR-7 could attenuate the activation of PI3K/AKT and MAPK signaling pathways [32-41], leading to suppression of tumor cell proliferation/survival and inhibition of tumor invasion/metastasis. Moreover, miR-7 could also restore the sensitivity of the therapy-resistant cancer cells to different therapies in several cancer types [42-46]. However, limited investigation of its effect on the proliferation of BRAFi-resistant melanoma cells has been reported. In our studies we first used TargetScan and PicTar to screen for candidate genes of miR-7 and compared these candidate genes with those genes that are already known to be involved in the resistance to BRAFi. We next confirmed that the over-expressions of EGFR, IGF-1R and CRAF were closely associated with resistance to BRAFi in VemR A375 melanoma cells. Introduction of miR-7 could decrease the expression levels of EGFR, IGF-1R and CRAF *in vitro* as well as in VemR A375 melanoma xenograft mice models, which indicated that EGFR, IGF-1R and CRAF were the target genes of miR-7 that are closely associated with the acquired resistance to BRAFi in VemR melanoma.

Another important question is whether miR-7 reverses the resistance to BRAFi in melanoma by modulating the expression of EGFR/IGF-1R/CRAF and the activity of their down-stream signaling pathways. Our studies indicated that miR-7 could decrease the expression of EGFR, IGF-1R and CRAF and partly reverse the resistance to BRAFi in VemR melanoma cells, further suppressed the tumor growth in VemR melanoma xenografted mice model. Despite their up-regulated expressions in VemR melanoma cells and definite associations with BRAFi resistance, EGFR, IGF-1R and CRAF might play different roles in development of resistance to BRAFi. This could be explained by their discrepant effects on the proliferation of VemR A375 melanoma cells and/or the activation of MAPK and PI3K/AKT pathways. Therefore, a further understanding of the underlying mechanisms and different roles of EGFR, IGF-1R and CRAF in BRAFi-resistant melanoma is a necessity. In addition, since other tumor suppressors/ oncogenes and associated signaling pathways also participate in the development of BRAFi resistance, such as NRAS, PTEN, cyclin D1, c-MET, etc., this may explain why miR-7 can only partially reverse BRAFi resistance. It is noteworthy that PTEN, a tumor suppressor and the most important negative regulator of PI3K/AKT signaling pathway, was also down-regulated in VemR A375 cell as compared with parental A375 cells by qRT-PCR analysis (Supplementary Figure S5D). TargetScan and PicTar anaysis also predicted that PTEN could be a potential target gene of miR-7. Our additional study further demonstrated that the expression level of PTEN mRNA was significantly increased after transfection with miR-7 for 48 hrs (Supplementary Figure S5E). In addition, we also showed that transfection with miR-7 mimics could significantly suppress the invasion and migration of VemR A375 cells as compared with transfection with miR-7 inhibitor by transwell migration and invasion assays (Supplementary Figure S5A, S5B and S5C). Since PTEN also plays an important role in tumor cell migration and spreading, we speculated that miR-7 could not only reverse the resistance of BRAF inhibitors, but also suppress the invasion and migration of VemR A375 cells through up-regulating the expression of PTEN. Nevertheless, these findings only revealed the potential interaction of these two molecules to some extent, further investigation is still needed to explore the underlying molecular mechanism of miR-7 in the regulation of PTEN expression and the subsequent changes of the related signaling pathways.

On the basis of the relative changes as assessed by qRT-PCR assays, we show that several of the dysregulated miRNAs identified in VemR melanoma cells have been reported to have a known association with BRAFi or chemotherapy resistance in many cancers including melanoma. For example, up-regulated miR-514 not only regulates the expression of NF1, a well-known tumor-suppressor gene in melanoma, but also contributes to altered BRAFi sensitivity *in vitro* [31]. In addition, down-regulated miR-17, 20a and 20b identified in VemR melanoma cells, have also been reported to be associated with chemotherapy resistance in non-small cell lung cancer [47]. Although these dysregulated miRNAs did not show any significant inhibitory effect on the proliferation of VemR melanoma cells, they might influence other biological properties of these resistant cells such as angiogenesis, invasion, metastasis, or tumor-related immune responses. Thus, further investigation is also of great importance for these dysregulated miRNAs to achieve an in-depth understanding of their potential roles in the pathophysiological changes of BRAFi-resistant melanoma.

In summary, our results demonstrated for the first time that miR-7 expression was decreased in both VemR A375 and Mel-CVR melanoma cells and its low expression contributed to BRAFi resistance. Furthermore, by decreasing the expression levels of EGFR, IGF-1R and CRAF, miR-7 could inhibit the activation of RAS/RAF/MEK/ERK (MAPK) and PI3K/AKT pathway and partially reverse the resistance to BRAFi in VemR A375 melanoma cells. Thus, it could advance the field and provide the basis for further studies in BRAF inhibitor resistance in melanoma.

## MATERIALS AND METHODS

### Cell culture and compounds

Cell lines were cultured in DMEM (Gibco BRL Co.Ltd., NY, USA) with 10% fetal bovine serum (Gibco BRL Co.Ltd., NY, USA). Cells were cultured in a 37 °C incubator with 5% CO_2_. Stocks of vemurafenib, linsitinib, gefitinib and GW5074 (Selleck Chemicals, Houston, TX) were prepared in DMSO. Resistant cell lines were generated by treating parental BRAFV600-mutant melanoma cell lines A375 and Mel-CV with increasing concentrations of vemurafenib. Cells with the ability to grow in 2 uM of vemurafenib were obtained 6 months after the initial drug exposure. Resistant lines were maintained in the continuous presence of 2 uM of vemurafenib, supplemented every 72 hrs.

### Microarray analysis

miRNA screening was carried out as described previously in duplicate for parental A375 and vemurafenib resistant A375 melanoma cells. Total mRNA was isolated from cell specimens and the miRNA expression profiling was performed by using Agilent Human miRNA V19.0 arrays at Ebioservice, Inc (Shanghai, China). A 2-tailed, 2-sample *t* test was used with differences of 2-fold or greater between samples (*p* < 0.05) regarded as significant.

### qRT-PCR

Total-RNA was extracted using TRIzol reagent (Invitrogen, Carlsbad, CA) according to the manufacturer's protocol. Briefly, total RNA was isolated using a column-based method and quality was assessed with an Agilent 2100 Bioanalyzer (Agilent Technologies, CA, USA). In all, 1 ng of total RNA was reverse transcribed using ABI Taqman miRNA assays (Applied Biosystems, Austin, TX, USA) and 3 ul of the product was used in subsequent Taqman PCR amplification, performed in triplicate on a validated real-time thermocycler (ABI 7900HT). Ct values were compared with standard curves generated with synthetic miRNAs to obtain absolute copy number per ng RNA, and values were normalized using stably expressed RNU44 small nucleolar RNA. mRNA expression analysis was conducted by quantitative PCR using SYBR green dye, with relative changes calculated by the Ct method.

### Pharmacological growth inhibition assays

Cultured cells were seeded into 96-well plates (1,000 cells per well) and 24 hrs after seeding serial dilutions of the relevant compound prepared in media were added. Cells were incubated for 72 hrs, and cell viability was measured using a CCK8 (Cell Counting kit-8) kit (Dojindo Laboratories, Kumamoto, Japan) according to the manufacturer's protocol. Briefly, 10 μl CCK8 solution (Dojindo, Kumamoto, Japan) was added to each well, and the samples were incubated at 37°C for 2 hrs before the absorbance was measured at 450 nm wave length. Viability was calculated as a percentage of control (DMSO-treated cells) after background subtraction, and all experiments were performed at least three times.

### miRNA and siRNA transfection

50-70% confluent cells were transfected with human miR-7 mimics or negative control (miR-NC) (GenePharma RNAi Company, Shanghai, China) and 30-40% confluent cells were transfected with EGFR/IGF-1R/CRAF siRNA or siRNA-NC (GenePharma RNAi Company, Shanghai, China) by Lipofectamine 2000 (Invitrogen, Carlsbad, CA) according to the manufacturer's protocol. Total RNA was extracted 24 hrs after transfection, and total cell protein were extracted 48 or 72 hrs after transfection.

### Western blotting

Total cellular proteins were extracted at 4 °C using RIPA lysis buffer containing protease and phosphatase inhibitors (Roche, Basel, Switzerland). Proteins (40 ug) were resolved on 10% SDS-PAGE and transferred to Immobilon-P membranes (Millipore, Bedford, MA). Western blots were probed with antibodies against p-ERK, t-ERK, p-AKT, t-AKT, EGFR, IGF-1R, CRAF (Cell Signaling Technology, Danvers, MA, USA). Horseradish peroxidase-coupled secondary antibodies (Jackson ImmunoResearch, West Grove, PA, USA) were diluted 1:5000 in phosphate-buffered saline containing 0.1% Tween-20 with 5% non-fat milk and visualized using enhanced chemiluminescence.

### Mouse model and immunohistochemistry (IHC)

Five after transfection of miR-7 mimics or miR-NC vemurafenib resistant A375 cells, the cells were trypsinized, rinsed, and subcutaneously implanted (2 × 10^5^ cells in 100 ul PBS) per flank of nude BALB/c mice (4-week-old males). One week later, the mice were sacrificed and the tumors were removed. The fresh tissues were quickly fixed with formaldehyde and processed to conventional hematoxylin and eosin (HE) staining, and the tumors were also processed to examine the expression of EGFR, IGF-1R, CRAF, pERK and pAKT by IHC. Cross-sectional images of tissue after microtome were observed and recorded through an optical microscope (Olympus, Japan). The total integrated optical density (IOD) was determined using an image-analysis program (Image pro-plus 6.0). Under a magnification of x200, three images were examined in each immunostained section and the average IOD was calculated.

### Statistical analysis

All measurements, including cell counting and qRT-PCR, and immunohistochemistry were carried out in triplicate and the values are expressed as the mean±SD; *P* values were calculated by using the Student's t test and values of *p* <0.05 were regarded as significant.

## SUPPLEMENTARY MATERIAL FIGURES AND TABLES


