# Supplementary material for: miR-7 reverses the resistance to BRAFi in melanoma by targeting EGFR/IGF-1R/CRAF and inhibiting the MAPK and PI3K/AKT signaling pathways
Source: Oncotarget. 2016 Jul 18;7(33):53558–70. doi: 10.18632/oncotarget.10669 (PMC5288205; doi:10.18632/oncotarget.10669)
Supplement: Supplementary file 1 [file oncotarget-07-53558-s001.pdf]

miR-7 reverses the resistance to BRAFi in melanoma by targeting EGFR/IGF-1R/CRAF and inhibiting the MAPK and PI3K/AKT signaling pathways

SUPPLEMENTARY FIGURES AND TABLES

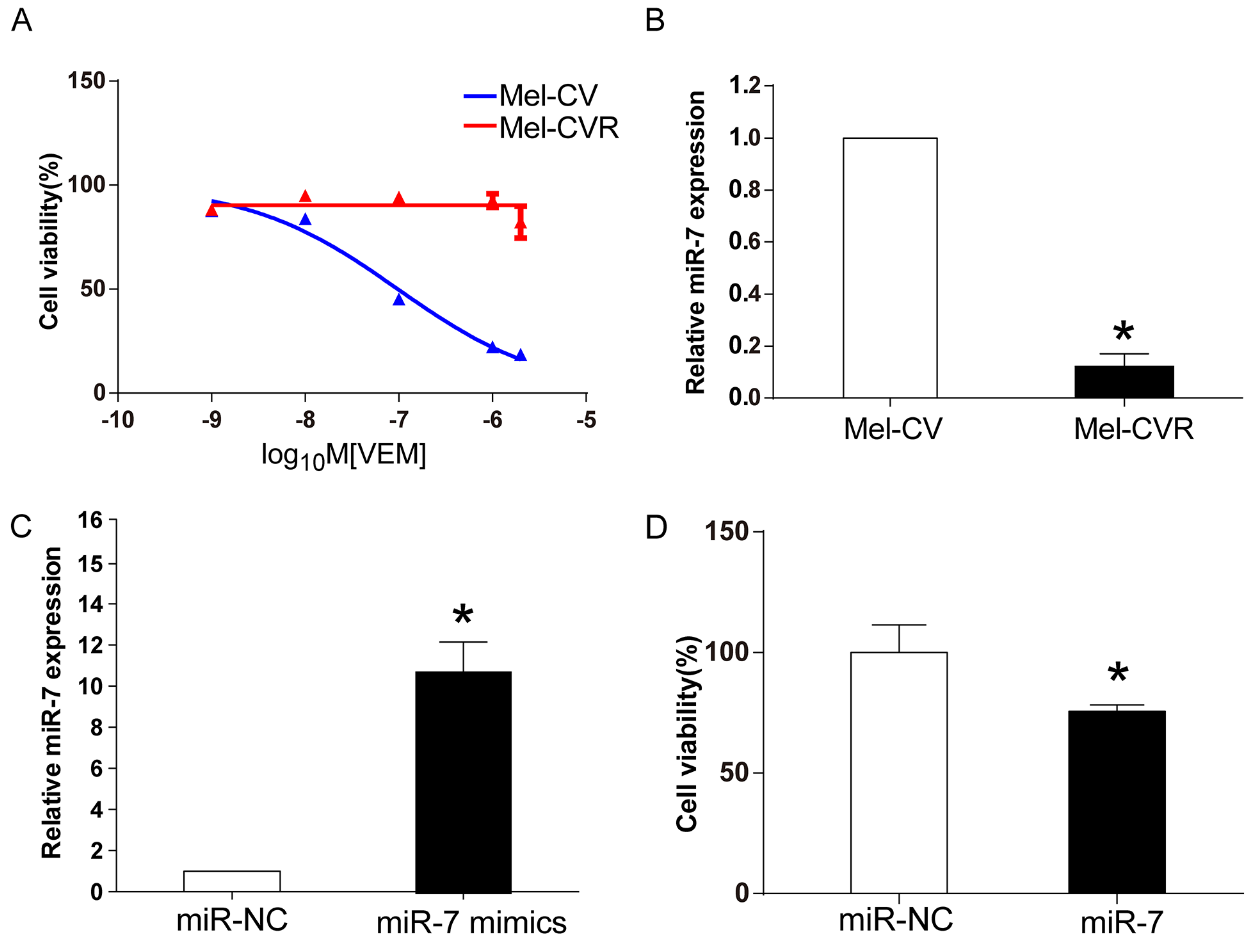

Supplementary Figure S1: A. Cell viability of parental Mel-CV melanoma cells (blue) and Mel-CVR melanoma cells (red) under different concentrations of vemurafenib treatment was assessed by CCK8 assays for 72 hrs. **B.** qRT-PCR showing down-regulated miR-7 expression in Mel-CVR melanoma cells compared with that in parental Mel-cv melanoma cells. The data were normalized to the level of U6 RNA in each sample. **C.** Expression levels of miR-7 in Mel-CV melanoma cells transfected with miR-7 or miR-NC. **D.** Cell viability of Mel-CVR melanoma cells stably transfected with miR-NC or miR-7 for 72 hrs. These experiments were carried out in triplicate and results are shown as the mean ± SD. *$p < 0.05$.

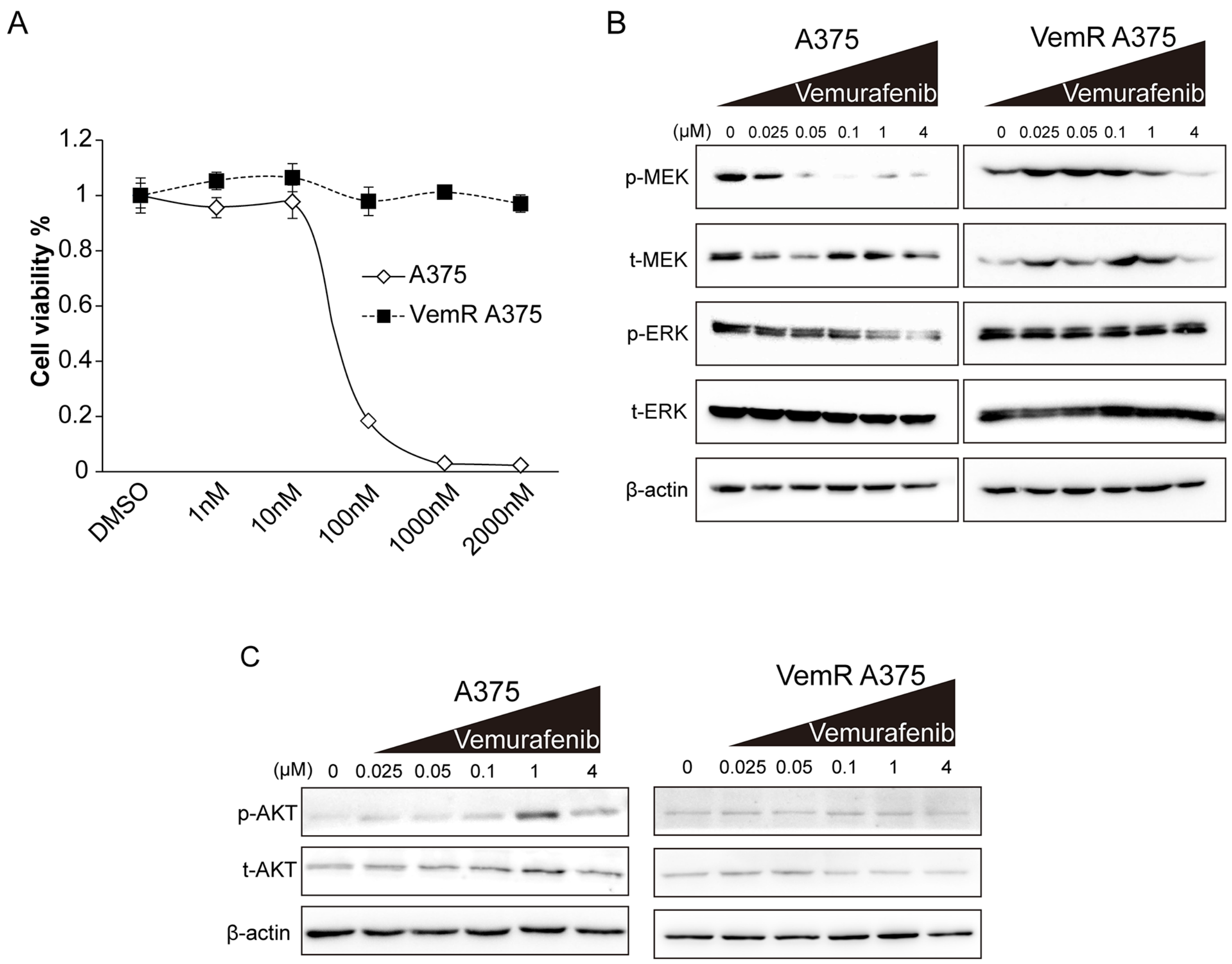

Supplementary Figure S2: A. Cell viability of parental A375 melanoma cells (solid) and VemR A375 melanoma cells (dashed) under different concentrations of vemurafenib treatment was assessed by CCK8 assays for 72 hrs. **B.** Expressions of MAPK pathway components and effectors in parental and VemR A375 melanoma cells treated with the indicated concentrations of vemurafenib for 72 hrs. **C.** Levels of AKT phosphorylation in parental and VemR A375 melanoma cells treated with the indicated concentrations of vemurafenib for 72 hrs.

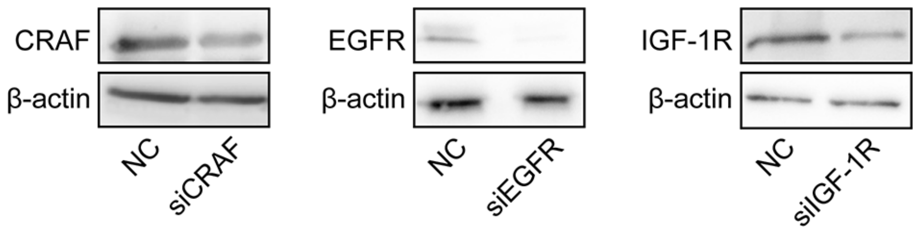

Supplementary Figure S3: The expression levels of EGFR, IGF-1R and CRAF protein in VemR A375 melanoma cells transfected with EGFR siRNA, IGF-1R siRNA, CRAF siRNA, respectively. Protein levels were assessed by western blotting assay 48 hrs after transfection.

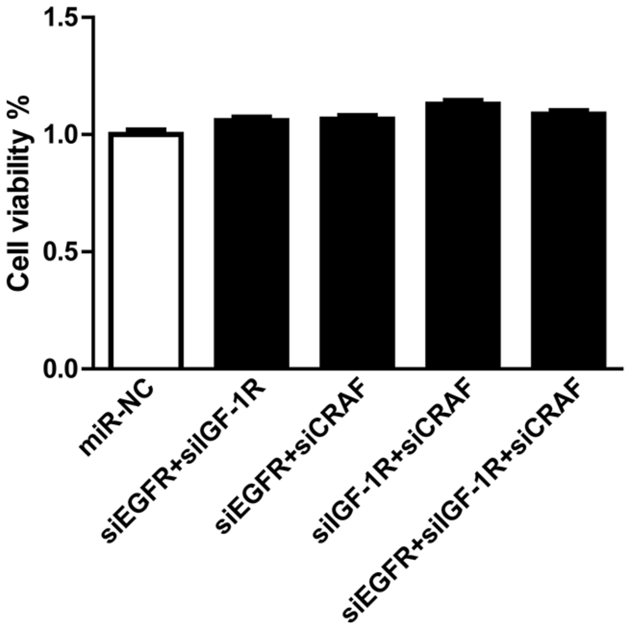

Supplementary Figure S4: Knock-down studies of up-regulated EGFR/IGF-1R/CRAF on VemR A375 melanoma cell growth exposed to vemurafenib. VemR A375 cells were transfected with siRNA normal control, EGFR siRNA/CRAF siRNA, EGFR siRNA/IGF-1R siRNA, IGF-1R siRNA/CRAF siRNA, EGFR siRNA/IGF-1R siRNA/CRAF siRNA, respectively. Cell viability was assessed by CCK8 assays 48 hrs after transfection. These experiments were carried out in triplicate and results are shown as the mean ± SD.

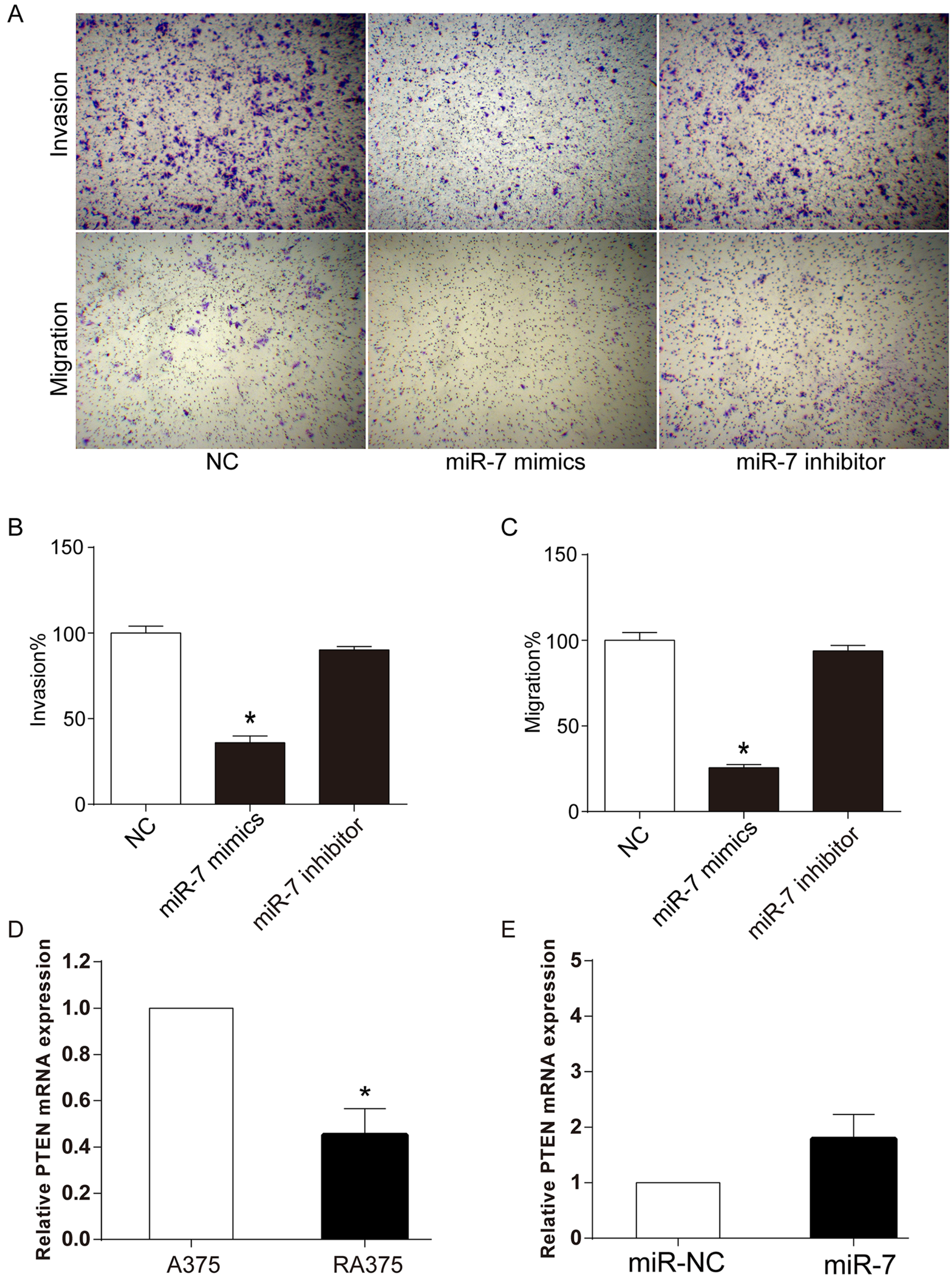

Supplementary Figure S5: Transwell assays A. and statistical analysis **B.** and **C.** of the invasion and migration of VemR A375 melanoma cells transfected with miR-7 mimics and miR-7 inhibitor for 48 hrs (for invasion analysis) and 72 hrs (for migration analysis). **D.** The expression level of PTEN mRNA of VemR A375 cell as compared with parental A375 cells by qRT-PCR analysis. **E.** The expression level of PTEN mRNA in VemR A375 cell after transfection with miR-7 for 48 hrs. These experiments were carried out in triplicate and results are shown as the mean ± SD. *$p < 0.05$.

Supplementary Table S1: Fold changes and *p* values of 17 dysregulated miRNAs identified in VemR A375 melanoma cells

Systematic Name	*p* values	Fold change
hsa-miR-129-1-3p	0.000043323	87.05416493
hsa-miR-17-5p	0.027173461	0.311453203
hsa-miR-18a-5p	0.028648788	0.190713147
hsa-miR-1915-3p	0.017090776	3.237515942
hsa-miR-19a-3p	0.028141265	0.280059306
hsa-miR-19b-3p	0.016703536	0.347395798
hsa-miR-20a-5p	0.013823847	0.318167149
hsa-miR-20b-5p	0.044842004	0.298200202
hsa-miR-3960	0.012644609	4.332473752
hsa-miR-4281	0.036456881	2.554409705
hsa-miR-4634	0.043401442	2.041951095
hsa-miR-509-3p	0.000512	83.48684077
hsa-miR-514a-3p	0.00031899	116.198628
hsa-miR-6090	0.018206656	3.099290217
hsa-miR-629-3p	0.024461546	22.48506535
hsa-miR-7	0.017548719	0.024831145
hsa-miR-937-5p	0.01962145	4.617689289

Supplementary Table S2: The sequences of other 16 dysregulated miRNAs identified in VemR A375 melanoma cells

Gene name	Sequence	
	Sense (5'-3')	Antisense (5'-3')
hsa-mir-17-5p mimics	CAAAGUGCUUACAGUGCAGGUAG	ACCUGCACUGUAAGCACUUUGUU
hsa-mir-18a-5p mimics	UAAGGUGCAUCUAGUGCAGAUAG	AUCUGCACUAGAUGCACCUUAUU
hsa-mir-19a-3p mimics	UGUGCAAAUCUAUGCAAAACUGA	AGUUUUGCAUAGAUUUGCACAUU
hsa-mir-19b-3p mimics	UGUGCAAAUCCAUGCAAAACUGA	AGUUUUGCAUGGAUUUGCACAUU
hsa-mir-20a-5p mimics	UAAAGUGCUUAUAGUGCAGGUAG	ACCUGCACUAUAAGCACUUUAUU
hsa-mir-20b-5p mimics	CAAAGUGCUCAUAGUGCAGGUAG	ACCUGCACUAUGAGCACUUUGUU
hsa-mir-129-1-3p inhibitor	AUACUUUUUGGGGUAAGGGCUU	
hsa-mir-514a-3p inhibitor	UCUACUCACAGAAGUGUCAAU	
hsa-mir-937-5p inhibitor	CCAGCCCCACCCUGACUCAC	
hsa-mir-3960 inhibitor	CCCCCGCCUCCGCCGCCGCC	
hsa-mir-4634 inhibitor	CCCCGGGCCGGUCGCGCCG	
hsa-mir-509-3p inhibitor	CUACCCACAGACGUACCAAUCA	
hsa-mir-629-3p inhibitor	GCUGGGCUUACGUUGGGAGAAC	
hsa-mir-1915-3p inhibitor	CCCGCCGCGUCGCCCUGGGG	
hsa-mir-4281 inhibitor	CCCCCCUCCCCGGGACCC	
hsa-mir-6090 inhibitor	GCCCCGCCCCUCGCUCCCC	
